# Dietary Fatty Acids at the Crossroad between Obesity and Colorectal Cancer: Fine Regulators of Adipose Tissue Homeostasis and Immune Response

**DOI:** 10.3390/cells10071738

**Published:** 2021-07-09

**Authors:** Manuela Del Cornò, Rosaria Varì, Beatrice Scazzocchio, Barbara Varano, Roberta Masella, Lucia Conti

**Affiliations:** Center for Gender-Specific Medicine, Istituto Superiore di Sanità, 00161 Rome, Italy; rosaria.vari@iss.it (R.V.); beatrice.scazzocchio@iss.it (B.S.); barbara.varano@iss.it (B.V.); roberta.masella@iss.it (R.M.)

**Keywords:** diet, inflammation, immune cells, fatty acids, adipose tissue, obesity, colorectal cancer, transcription factors

## Abstract

Colorectal cancer (CRC) is among the major threatening diseases worldwide, being the third most common cancer, and a leading cause of death, with a global incidence expected to increase in the coming years. Enhanced adiposity, particularly visceral fat, is a major risk factor for the development of several tumours, including CRC, and represents an important indicator of incidence, survival, prognosis, recurrence rates, and response to therapy. The obesity-associated low-grade chronic inflammation is thought to be a key determinant in CRC development, with the adipocytes and the adipose tissue (AT) playing a significant role in the integration of diet-related endocrine, metabolic, and inflammatory signals. Furthermore, AT infiltrating immune cells contribute to local and systemic inflammation by affecting immune and cancer cell functions through the release of soluble mediators. Among the factors introduced with diet and enriched in AT, fatty acids (FA) represent major players in inflammation and are able to deeply regulate AT homeostasis and immune cell function through gene expression regulation and by modulating the activity of several transcription factors (TF). This review summarizes human studies on the effects of dietary FA on AT homeostasis and immune cell functions, highlighting the molecular pathways and TF involved. The relevance of FA balance in linking diet, AT inflammation, and CRC is also discussed. Original and review articles were searched in PubMed without temporal limitation up to March 2021, by using fatty acid as a keyword in combination with diet, obesity, colorectal cancer, inflammation, adipose tissue, immune cells, and transcription factors.

## 1. Introduction

The global incidence of overweight/obesity is expected to reach 20% by 2025 and represents a major health problem, afflicting currently adults and children worldwide [1]. It represents a considerable cost to public health and a clinically urgent issue for the population in several countries [2,3]. Excess adiposity is associated with increased incidence of several cancers, including colorectal cancer (CRC), and represents an important indicator of survival, prognosis, recurrence, and response to therapy. CRC is the third most common cancer worldwide and a leading cause of death with burden expected to increase in the coming years (IARC 2020. Colorectal cancer. Source: Globocan. The Global Cancer Observatory. Available from: http://gco.iarc.fr/today, accessed on 30 May 2021). Both genetic background and a range of modifiable environmental/lifestyle factors play a role in CRC aetiology. Specifically, enhanced adiposity, particularly abdominal obesity, is associated with increased CRC incidence, and cancer risk is highly modifiable by diet. White adipose tissue (AT), where diet-delivered signals converge, is now recognized as the largest endocrine organ and plays a key role in metabolic and immune homeostasis [4]. To date, several studies have investigated the role of diet/nutrition in CRC showing both a causal and protective role in tumour development [5].

In addition of being an established risk factor for CRC [6], enhanced adiposity is also associated with worse outcomes [7,8], although the detrimental relationship between obesity and CRC is complex and not yet precisely defined. In this regard, it has been postulated that the production, by AT, of a large spectrum of adipocytokines and metabolites, showing proinflammatory and cancer prone features, is of great importance. Furthermore, obesity-related metabolic alterations (i.e., metabolic syndrome, insulin resistance, lipid metabolism impairment, endocrine changes and oxidative stress) may promote CRC occurrence and progression [9].

Diet and excess adiposity can also affect cancer development by influencing tumour surveillance and shaping the host immune response [10]. Indeed, white AT is now recognized as the largest endocrine organ where signals from diet converge, playing a key role in both metabolism and immune system homeostasis [9,11]. Furthermore, a whole set of immune cells, with either proinflammatory (i.e., dendritic and mast cells, M1 macrophages, neutrophils, Th1 CD4 and CD8 T lymphocytes, and B cells) or antiinflammatory (i.e., M2 macrophages, regulatory T (T_reg_) cells and Th2 CD4 T lymphocytes, and eosinophils) properties, is held in AT, whose polarization profile depends on the health status of adipocytes [12,13].

Growing evidence indicates that a chronic low-grade inflammatory state, called “meta-inflammation”, occurs in metabolically active tissues, including AT, and characterizes obesity, thus contributing to the impairment of immune cell functions, and representing a key determinant in the development of obesity-related morbidities, including CRC [14]. Indeed, the molecular mechanisms underlying inflammation-promoted tumorigenesis or tumour progression have become an important topic in cancer research, and the crucial importance of the AT microenvironment in regulating the dynamic interplay between neoplastic and immune cells has recently emerged [15].

In this regard, fatty acids (FA), introduced with the diet and processed/released by AT, are gaining importance as main actors in this interplay for their capacity to influence both cancer cell proliferation and the host immune response [16]. Depending on their chemical features, FA can exhibit either pro- or anti-inflammatory activity [17,18]. In general, long-chain saturated fatty acids (SFA) have been associated with inflammatory effects while short-chain fatty acids, derived from microbial fermentation of indigestible foods, exert anti-inflammatory actions [19]. Likewise, ω6 and ω3 polyunsaturated fatty acids (PUFA) have been associated with inflammatory or anti-inflammatory pathways, respectively [20]. This is accomplished through several mechanisms, by controlling the activation of either cell surface or intracellular receptors, and regulating signal transduction, gene expression, and the nuclear abundance of transcription factors (TF) [17,18,21]. By virtue of their properties, FA have the potential to control host surveillance mechanisms and shape anticancer responses by directly influencing both innate and adaptive immunity and by regulating AT immune and metabolic homeostasis.

The following sections provide an overview of the changes of FA profiles in human AT occurring in obesity and in CRC and of the role played by FA as regulators of inflammation and immune responses, focusing on the molecular mechanisms and TF involved. Non-homogeneous and sometimes contradictory results were found due to the different fat depots analysed or different FA doses and treatment times in intervention studies. Nevertheless, the type of effect (inflammatory versus anti-inflammatory) of specific FA classes was confirmed in all in vitro and in vivo studies. The potential of these molecules to act as a link among diet, AT inflammation, and CRC development is discussed.

## 2. Fatty Acid Profiles in Obesity and Colorectal Cancer and Their Relationship with Dietary Intake

The body fat mass is distributed in two main fat depots, visceral (VAT) and subcutaneous (SAT) white AT, whose anatomical distribution is influenced by several factors, including age, sex/gender, and nutrition [22]. When energy balance is shifted toward obesity, AT undergoes profound modifications such as adipocyte hyperplasia, tissue remodelling, changes in FA composition, inflammation, and metabolic dysfunction [23]. A preferential accumulation of SFA and monounsaturated fatty acids (MUFA) have been described for SAT and VAT, respectively, in obese individuals, despite a comparable PUFA composition [24,25]. However, the changes in FA composition occurring in obese subjects with respect to normal-weight individuals have been only poorly explored. We reported a marked decrease of the ω3/ω6 PUFA ratio in VAT of obese as compared to lean subjects, despite comparable total content of SFA, MUFA, and PUFA [26]. By analysing the ω6 PUFA family members, a selective increase of the proinflammatory arachidonic acid (AA) and parallel decrease of the anti-inflammatory γ linolenic acid (GLA) was unravelled in obese subjects [27]. Palmitoleic (POA) and stearic (SA) acids were also found enriched in SAT and VAT of obese versus lean individuals, in association with a higher stearoyl-CoA-desaturase 1 (*SCD1*) index [28,29]. Furthermore, an increase in circulating free FA have been reported in obesity as a consequence of enhanced release by the enlarged AT mass [30,31].

Although these findings suggest that the altered FA processing and conversion rate can contribute to changing FA profiles in obesity, there is strong evidence that FA content in AT mirrors their relative abundance in the diet. A significant correlation between dietary FA intake and FA composition of AT was demonstrated in obese individuals, in particular for oleic (OA), linolenic (LA), and α-linoleic (ALA) acids, and for total ω6 PUFA [32]. Interestingly, visceral obesity was positively associated with ω6 PUFA and inversely associated with MUFA and ω3 PUFA content in AT [25]. Furthermore, in a large Swedish cohort study, the relative proportion of some PUFA (i.e., LA, ALA, eicosapentaenoic acid, EPA, and docosahexaenoic acid, DHA), MUFA (i.e., POA), and SFA (i.e., palmitic acid, PA) in SAT was found to reflect their dietary intake [29,33,34]. Accordingly, we recently reported that the higher AA content detected in the VAT of obese subjects is associated with a higher intake [27,29]. Similarly, higher levels of OA were found in SAT of overweight subjects consuming MUFA-compared to SFA-rich diets [35]. Thus, changing the nature of the fat consumed may alter FA composition of AT and has a profound influence on the type of FA available to the body [36]. Furthermore, unhealthy dietary habits may influence AT-associated and circulating FA profiles contributing to the alteration of metabolic pathways [29,34]. Under obese conditions, AT also expresses high levels of FA synthase, an enzyme responsible for the synthesis of FA from dietary carbohydrates, which in turn induces inflammation [37].

A number of studies analysing the AT composition in CRC-affected individuals have described alterations of FA metabolism and profiles in different fat depots and specific FA profiles have been correlated with the risk of developing CRC [38,39].

As reported for obese subjects, an unbalanced ω3/ω6 PUFA ratio and accumulation of proinflammatory ω6 PUFA (mostly dihomo-γ-linolenic acid DGLA and AA) in AT take place also in CRC subjects, even though differences were described depending on the relative abundance of individual PUFA and fat depots involved [27,40,41,42]. Specifically, by comparing VAT and SAT FA profiles in colon cancer (CC) patients it was evidenced that FA composition of SAT is only slightly affected, while a significant decrease of the ω3 PUFA ALA and stearidonic acid (SDA), along with increased DGLA and AA content, occurs in VAT [41]. Changes in the ω3/ω6 PUFA profile (higher DGLA and docosapentaenoic acid, DPA, versus lower ALA) were conversely reported in SAT from CRC patients in a different study, in association with markers of systemic inflammation [42]. Moreover, an increase of total SFA and MUFA content was associated with CRC [41]. In contrast, some old studies failed to reveal any change in the relative abundance of the different FA in cancer subjects [43,44].

Furthermore, we reported alterations in VAT FA profiles in CRC, highlighting differences between lean and obese patients [26,27,29]. Specifically, we evidenced a decrease in the ω3/ω6 PUFA ratio and an increased content of DGLA and docosatetraenoic acid (DTA) in cancer patients, irrespective of body weight [26,27]. However, when obese and normal weight patients were compared, accumulation of AA was selectively observed in obese CRC subjects with respect to healthy individuals. Conversely, lean patients were found to be characterized by reduced SFA content despite a higher dietary intake [27,29]. The enrichment in proinflammatory FA observed in cancer patients is associated with constitutive signal transducer and activator of transcription (*STAT)-3* activation in adipocytes and enhanced release of inflammatory cytokines and chemokines [27].

Changes in lipid metabolism in AT has also been related to tumour progression [45]. In particular, MUFA content in visceral peritumoral fat of CRC patients was associated with advanced disease, whereas similar levels of SFA and PUFA were found irrespective of the tumour stage [45]. At the same time, the SAT content of MUFA has been negatively associated to the risk of developing CC in epidemiological studies [46], highlighting that the same category of FA (e.g., MUFA) can play different roles in cancer onset/progression depending on the timing and type of AT [45,46].

As with obese individuals, the levels of circulating free FA have been recently found to be increased in CRC patients and associated with cancer risk [47]. Conversely, CRC risk has been inversely correlated to dietary PUFA intake. A growing body of epidemiological evidence has linked to ω3 PUFA-rich diets or ω3 PUFA dietary supplementation to a potential lower risk of CRC, and a recent study definitely correlated fish intake and dietary intake of individual and total ω3 PUFA with lower incidence of CRC [48].

In conclusion, the altered ω3/ω6 PUFA balance in AT and the enrichment in SFA reported in most studies seems to be a common feature of obese and CRC-affected subjects. This could markedly affect the function of AT and distal tissues, such as the intestinal epithelium, as a result of an increased ω6 PUFA-mediated inflammation and a reduced protective effect of ω3 PUFA. The changes in FA profiles in different fat depots sustain proinflammatory microenvironment in CRC patients, supporting a role for both unbalanced dietary intake and alterations in FA metabolism and storage in colorectal tumorigenesis.

## 3. Fatty Acids and Adipose Tissue Homeostasis

The importance of AT in controlling systemic inflammation has been pointed out in recent years. Due to its endocrine character, alterations in this tissue may lead to various metabolic disorders such as diabetes, cardiovascular and liver diseases, and cancer. The recognition that AT not only synthesizes and stores FA but also releases these compounds, together with a large number of other active factors able to act in an autocrine and paracrine manner [13], has provided a conceptual framework, which helps to understand how unhealthy diets and obesity contribute to the development of several disorders affecting distal organs and tissues.

People with obesity exhibit a general proinflammatory profile. Changes of cytokine/adipokine secretion by adipocytes and free FA release by AT couple with dramatic changes in the immune cell repertoire and function shifting the balance of cell subsets and soluble mediators toward a proinflammatory profile [49,50,51]. This results from an altered balance of key transcription factors able to promote inflammation through the induction of molecules such as *TNFα*, *IL-6*, *IL-1β*, and Toll-like receptor (*TLR*) 4. These, in turn, exacerbate the inflammatory state [52,53,54] and can promote a favourable microenvironment to CRC onset/evolution [55].

SFA have been shown to negatively affect metabolic functions [56] and to activate inflammatory pathways by acting as ligands of receptors, such as the *TLR*, involved in the innate immune response. Conversely, endogenous or dietary PUFA are precursors of both pro- and anti-inflammatory lipid mediators [57].

Studies carried out on in vitro models have evidenced the ability of SFA to induce an inflammatory response in AT through the *TLR/NF-κB* pathways. At the same time, adipocytes from SAT and VAT of obese subjects, treated with free FA (i.e., OA, LA, AA, lauric and myristic acids or PA and SA mixtures) showed a proinflammatory cytokine profile [58,59]. Conversely, ω3 PUFA, in particular DHA and EPA, were reported to exert an anti-inflammatory role in whole SAT and VAT and in isolated adipocytes from obese subjects by reducing proinflammatory mediators [60,61,62]. These data are in line with our studies demonstrating the capacity of DHA to downregulate *STAT3* activation and IL-6 secretion, and to increase adiponectin expression, in VAT adipocytes [26,27]. On the contrary, AA exposure results in a significant *STAT3* activation and concomitant downregulation of *PPAR-γ* expression [26,27]. The capacity of pro- and anti-inflammatory PUFA to regulate immune pathways in VAT suggests a role for the altered PUFA composition in shaping immune cell phenotypes in obesity [63]. Ω3- and ω6-PUFA, in particular DHA and AA, have also been demonstrated to differently influence the adipocyte transcriptional program in normal-weight and obese subjects [61,62,64], acting on genes involved in AT inflammation and metabolism [64,65]. The ability of dietary PUFA to regulate adipocyte gene expression further elucidates the role of diet in the modulation of AT inflammation, even though the mechanisms responsible of such an effect still need to be clarified.

Several intervention studies have also investigated the role of FA in the control of AT homeostasis. Discordant results have been reported mainly due to the dose and timing of FA supplementation, the different populations investigated, and differences related to the type of body fat depot analysed. Nevertheless, the beneficial role of ω3 PUFA consumption on a wide range of AT inflammatory responses have been undoubtedly highlighted. In a clinical trial of obese participants, ω3 PUFA-rich fish oil (FO) supplementation was found to reduce the expression of *NLRP3* inflammasome associated genes in AT and circulating IL-18 levels [62]. Accordingly, a meta-analysis including 17 studies revealed that FO supplementation is associated with increased insulin sensitivity among people with metabolic disorders [66], likely by decreasing the levels of *TNFα*, *IL-1β* and *IL-6*. The same conclusion was obtained in a pilot study carried out on 32 overweight and/or obese individuals with type 2 diabetes mellitus (T2DM) who received FO supplementation [67]. Among the genes modulated by the consumption of different PUFA sources are those involved in AT inflammation and metabolism, including inflammasome-associated *IL-18*, *IL-1β* and *IL-1RN*, and genes involved in impaired fasting glucose in obese subjects [62,68]. Moreover, in a clinical trial (FFAME) involving healthy volunteers and based on EPA and DHA supplementation, ω3 PUFA showed immune-modulatory and anti-inflammatory action during AT inflammation induced by experimental endotoxemia [61,69]. Changes in the expression of genes related to inflammation and the immune response were also observed in SAT from overweight/obese women supplemented with EPA and/or lipoic acid and subject to weight loss and dietary interventions [70]. Conversely, no effect on SAT inflammatory genes was exerted by DHA supplementation in obese postmenopausal women [71].

A strong association between AT inflammation and its SFA and MUFA content was also demonstrated [24]. KOBS study carried out on obese individuals showed that surgery-induced weight loss reduces the expression of inflammatory pathways (*IL-1β* and *NF-κB*), and unravelled a positive/negative association between inflammation and SFA and MUFA content, respectively, in both SAT and VAT [20]. *FADS1/FADS2* genotypes were found able to modify these correlations, both in KOBS and DiOGenes studies, indicating that variants in this gene cluster may influence the interaction between AT FA and tissue inflammation [24,72].

## 4. Fatty Acids and Immune Cells: Regulation of Transcription Factors, Inflammatory Pathways, and Effector Functions

Obesity is associated with increased numbers and activation levels of specific immune cell subsets responsible for skewing the balance towards a proinflammatory status. AT-associated active molecules, including FA, may represent important determinants in shaping the immune cell phenotype and tissue microenvironment. In particular, the balance of saturated/unsaturated FA and the relative composition of ω3/ω6 PUFA may have significant consequences on immune system homeostasis, acting as an important link between unhealthy dietary habits/obesity and impaired cancer surveillance [73,74,75].

Although only a few studies have focused on FA-mediated immune cell regulation in the context of human AT, the immunomodulatory effects of dietary FA on cells of both innate and adaptive immunity have been investigated in a number of in vivo studies and in in vitro and animal models [76]. The molecular mechanisms and pathways involved are still poorly understood and often present cell type-specific features.

In this section we discuss the human studies that highlighted the immunomodulatory properties of FA following their dietary supplementation and after in vitro/ex-vivo stimulation of immune cells, focusing on the molecular pathways involved.

### 4.1. Effects of Dietary Fatty Acid Supplementation on Blood Immune Cell Gene Expression Profiles

The role of FA in the regulation of immune/inflammatory responses has been investigated in several intervention studies aimed at assessing the effects of consumption of different FA on inflammation-related genes. As discussed above, the analysis of inflammatory response gene expression profiles in AT have generated discordant results mainly due to differences related to the type of body fat depot analysed. Conversely, gene expression profiling of circulating immune cells within the peripheral blood mononuclear cell (PBMC) fraction turned out to have the potential to clarify the molecular effects of FA consumption on human health [77]. In dietary intervention studies, PBMC gene expression profiles have been shown to act as potential biomarkers reflecting metabolic changes in the liver and AT [78,79]. Furthermore, they can reflect metabolic changes due to long-term nutritional adaptation thus mirroring the individual physiologic or pathologic state [78].

In recent studies exploiting whole genome transcriptional approaches on PBMC (Table 1), annotation and pathway analysis have shown that the supplementation of ω3 PUFA-rich oils modulates the expression of genes involved in inflammatory pathways, such as eicosanoid synthesis, interleukin signalling, oxidative stress response, cell cycle, cell adhesion, apoptosis, scavenger receptor activity, and DNA damage, thus confirming and shedding further light on the anti-inflammatory/antioxidant role of dietary ω3 PUFA [80,81]. Conversely, SFA intake was confirmed to be associated with postprandial upregulation of genes involved in proinflammatory pathways [17].

More generally, high-MUFA and high-ω3 PUFA diets result in anti-inflammatory PBMC gene profiles, or at least less pronounced proinflammatory responses as compared to SFA consumption.

Specifically, the effects of ω3 PUFA intake on PBMC gene expression have been investigated in two studies including either metabolically healthy overweight and obese individuals or insulin resistant obese subjects [82,83]. The supplementation for 6–8 weeks resulted in consistent changes in the oxidative stress response mediated by erythroid-derived nuclear factor like 2 (*NRF2*), *PPAR-α*, hypoxia inducible factor (*HIF*), and *NF-κB* signalling pathways [82,83]. Interestingly, the anti-inflammatory effects of ω3 PUFA on circulating immune cells were gender-related and were observed despite any change in serum inflammatory markers [82]. Conversely, a longer (three-months) supplementation with ω3 PUFA in women with obesity was found to decrease the plasma concentrations of several inflammatory markers [84]. Contextual microarray analysis of PBMC demonstrated positive effects on αα target genes related to lipid metabolism and on *NRF2*-regulated antioxidant enzymes [84]. In line with this evidence, negative effects of fish and vegetable oil (ω3 PUFA- and MUFA-rich, respectively) supplementations on PBMC inflammatory pathways have been also demonstrated in elderly individuals [86], in subjects with Alzheimer’s disease [85], and in a cohort of women with polycystic ovary syndrome [87], thus further highlighting the role of ω3 PUFA intake in redirecting immune responses in conditions where chronic inflammation plays a pathogenic role. Conversely, some other studies failed to demonstrate any change in the expression of inflammatory genes in PBMC after supplementation with either nutritional doses or high pharmacologic doses of EPA and DHA [88], or after fatty fish intake [68]. The gene expression program of healthy individuals is also strongly influenced by dietary FA intake. Several intervention trials with healthy lean subjects highlighted extensive gene expression modulation in PBMC following FO [89]. Among these genes are those involved in cell cycle and apoptosis [89], in glucose and β-oxidation [90] or in lipid metabolism and inflammation [86,90], and *NRF2* target genes exhibiting antioxidant properties [89].

Other dietary intervention studies based on vegetable oils, such as flaxseed oil and camelina sativa oil, which are enriched in ALA, have demonstrated anti-inflammatory effects on PBMC gene expression. In particular, a significant increase in *PPAR-γ* levels has been reported in individuals with impaired fasting glucose and in obese and T2DM subjects [68,92,93]. Furthermore, the expression of several genes involved in lipid metabolism and inflammation was favourably changed in PBMC when replacing SFA-rich with ω6 PUFA-rich diets [80].

According to the effects on gene expression described in most studies, ω3 PUFA supplementation was reported to shift the effector functions of specific immune cell populations within the exposed PBMC toward an anti-inflammatory profile [94,95]. Reduced natural killer cell activity and T lymphocyte proliferation in mitogen-stimulated PBMC was observed following a 12-week FO intake in healthy individuals [94]. A comparable negative effect on lymphocyte proliferation was observed following intake of moderate levels of the anti-inflammatory ω6 PUFA GLA [95], thus suggesting that these PUFA have the capacity to control excessive immune cell activation that could have deleterious effects and lead to immunosuppression. 

### 4.2. Effects of In Vitro Fatty Acid Exposure on Immune Cell Functions

A number of in vitro studies have confirmed the regulatory effects of ω3 PUFA on immune homeostasis/inflammation and have highlighted the capacity of the different classes of FA to modulate the biology of human cell populations participating to both innate and adaptive immune responses, and actively involved in tumour surveillance.

These studies (summarized in Table 2) had the advantage to directly relate the effects of single FA treatment to a specific cell subset and allowed an in-depth understanding of the molecular mechanisms involved. FA were shown to regulate different functions, depending on the cell type, through the modulation of gene expression and by regulating the activity of different families of TF, mainly including *PPAR* (α, β, and γ), *LXR, SREBP*, and *NF-κB* [96,97].

#### 4.2.1. Innate Immunity Cells

**Figure 1:**
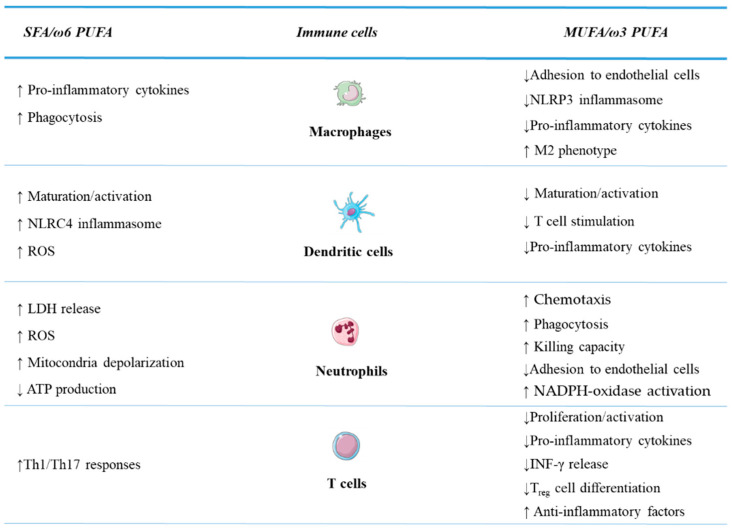
Effects of fatty acids on immune cell functions.

The immune cell populations involved in inflammatory response and contributing to the early defence against infections, tumours, and metabolic insults have been studied for their responsiveness to dietary FA. To date significant effects have been described for monocytes, macrophages, dendritic cells (DCs), and neutrophils and a number of functions have been identified to be differentially regulated by saturated and unsaturated FA. These include: (a) cell activation/maturation, (b) production and secretion of immune mediators, (c) phagocytic/cytotoxic activity, and (d) T cell stimulatory capacity (Figure 1).

Monocytic cell lines and primary monocytes exposed to free FA (mostly PA) were shown to exhibit an increased expression of inflammatory cytokines, which is mediated by activation of *MAPK* and the *NF-κB* canonical pathway [99,100,101,104,105] or *AP-1* [98,104]. Interestingly, PA, in combination with *TNFα*, stimulates a more marked increase in *CCL2* and *CXCL8* production as compared with either treatment alone, requiring the *TLR4/TRIF/IRF3* signalling cascade [102,103]. Since elevated levels of free PA and *TNFα* have been reported in obesity, their cross-talk may be a key driver of obesity-associated chronic inflammation via excessive chemokine production.

Similarly, PA was shown to stimulate the production of *IL-1β* in DC via *TLR4/MD-2*, through an inflammasome dependent mechanism, ultimately leading to *NF-κB* activation and reactive oxygen species (ROS) generation, and to cell maturation/activation [106]. Other studies, however, failed to demonstrate proinflammatory effects of SFA in DC [107,108]. Furthermore, although *TLR4* represents the main receptor involved in the recognition of SFA by myeloid cells [99,102,103,106], Snodgrass and co-authors have reported the capacity of PA to directly activate *TLR2,* highly expressed on human monocytes, by inducing heterodimerization with *TLR1* [105]. PA-dependent *TLR2* triggering leads to *NLRP3* inflammasome activation and *IL-1β* secretion in the THP-1 cell line, while the activation of this pathway does not occur following exposure to other dietary SFA such as myristate and laurate [98].

In vitro models have also been widely used to investigate the effects of FA on neutrophil homeostasis. According to their general proinflammatory activity, dietary SFA were reported to stimulate ROS production in neutrophils [109]. A comparable inflammatory response was generated by MUFA, in particular OA, which was found to enhance phagocytosis, killing capacity and cytokine production in these cells [109,112], and ω6 PUFA, specifically AA, able to enhance ROS production, LDH release, mitochondria depolarization, and lipid accumulation, while reducing ATP production [112,115,116,117]. Conversely, a different member of the ω6 PUFA family, linoleic acid (LA), exerts anti-inflammatory activity in neutrophils [119].

AA was also shown to maintain an inflammatory state in THP1 and primary monocytes by interfering with the *IFN-γ* signalling pathway, reducing the phosphorylation of the signal transducer and activator of transcription (*STAT*)-1, and ultimately blocking the immunosuppressive activity of indoleamine 2,3-dioxygenase (*IDO*) in vivo [118].

The impact of ω3 PUFA on the activation and cytokine/chemokine production of innate immunity cells, and the regulatory mechanisms behind it, have been also extensively investigated. According to the effects observed following dietary supplementation, ω3 PUFA, in particular DHA and EPA, have been reported to exert anti-inflammatory activities on in vitro stimulated monocytic cell lines. This was achieved either through inhibition of the *NF-kB* pathway leading to reduced expression of proinflammatory mediators [62,100,120,121], or via *p38 MAPK*-mediated activation of *KLF4,* resulting in M2 polarization [122]. Moreover, DHA and EPA were found to attenuate cytokine gene expression induced by LPS stimulation in THP-1- and primary monocyte-derived macrophages (MDM) in a synergistic manner [123], and the timing of PUFA stimulation turned out to play an important role [123].

The G protein-coupled receptors (*GPR*) have been characterized as the ω3 PUFA receptor/sensor in both macrophages and mature adipocytes, involved in mediating the anti-inflammatory effects of EPA and DHA [132]. By signalling through *GPR120* and GPR40, DHA has been shown to exert an anti-inflammatory activity on THP-1 and primary MDM through several mechanisms, including the inhibition of *NLRP3* inflammasome activation and prostaglandin E2 (PGE2) mediated signalling [120,124].

Similar to macrophages, the functional activation/maturation of DC is reduced following ω3 PUFA exposure [107,108,124,125]. This effect, however, is independent of *NF-κB* [108] and requires *PPAR-γ* [125] or *PPAR-γ:RXR* heterodimers [126]. In this regard, there is considerable evidence that ω3 PUFA modulate the transcription of genes involved in lipid metabolism and inflammation in DC by acting as ligands of *PPAR* [126] or by regulating the activity of liver X receptors (*LXR*). Focusing on cellular function, monocyte-derived DC (MDDC) cultures stimulated with DHA, EPA, and ALA exhibit a decreased proportion of activated *CD1*a+ cells, and reduced expression of *IL-6* and *GPR120* [127]. Additionally, DHA and EPA downregulate the expression of major histocompatibility complex class II (*MHC-II*) and costimulatory molecules on the surface of DC [107,108], and reduce the expression of *TNFα* and *IL-12p40* as compared to PA or OA [108], while MDDC differentiation in the presence of DHA results in increased expression of *MHC-II* and costimulatory molecules coupled to reduced secretion of both pro- and anti-inflammatory cytokines [126]. Other studies have reported the inhibition of cytokine production in EPA- and DHA-treated MDDC, coupled to reduced ability to stimulate activation, proliferation, and *IL-2/IFN-γ* production in T lymphocytes [108,125], thus suggesting that the immunomodulatory effects of dietary FA on DC may have a significant influence on adaptive immune responses.

ω3 PUFA were also reported to control inflammation in in vitro stimulated neutrophils, and the oxidation status of these FA proved essential in determining their effects. Indeed, oxidized EPA was found to significantly inhibit in vitro neutrophil and monocyte adhesion to endothelial cells by blocking endothelial adhesion receptor expression and by activating *PPAR-α* [128]. Interestingly, a proinflammatory effect of ω3 PUFA on neutrophils has also been reported [129,133]. Specifically, FA-mediated activation of *GPR84*, abundantly expressed in these cells, triggers inflammatory responses such as chemotaxis, ROS production, granule-localized receptor mobilization, and NADPH-oxidase activation [129]. The latter evidence indicates that different, and even opposite, responses can be stimulated by the same type of FA depending on the cell type, oxidation level, or receptor engaged.

Finally, saturated and unsaturated FA were also shown to differentially influence inflammatory gene expression in LPS-stimulated PBMC [130,131]. MUFA and ω3 PUFA reduce inflammatory gene expression, by influencing *NF-κB* cell availability and modifying its post-translational regulation. Conversely, SFA and ω6 PUFA elicit minor effects on inflammatory gene expression regardless of LPS stimulation [130].

#### 4.2.2. Adaptive Immunity Cells

According to what was reported for innate immunity cells, saturated and unsaturated FA were shown to exert opposite effects on signalling pathways, gene expression and effector functions of cells participating to adaptive immunity, mostly T lymphocytes (Figure 1). Exposure of naive T lymphocytes to PA was found to induce the expression of signalling lymphocytic activation molecule family member 3 (*SLAMF3*), a molecule found upregulated in T cells from T2DM patients and associated with their ability to produce *IFN*-γ and *IL-17* [111]. PA-induction of *SLAMF3* required the activation of *STAT5* and *PI3K/AKT* signalling pathway, in keeping with previous data showing the key role of this pathway in PA-induced Th1/Th17 cell commitment [134]. The elevated SFA levels in obesity and T2DM might contribute to the abnormal T lymphocyte activation and increased Th1/Th17 responses observed in these conditions. It is worth noting that Th17 cell-mediated responses have been found to promote colorectal tumour initiation and growth in preclinical models and are associated with worse outcomes in CRC patients [135].

Chronic inflammation in obesity and metabolic diseases also results from mitochondrial dysfunction and redox imbalance, and SFA have been postulated to control these mechanisms. Stimulation of PBMC from healthy lean donors with PA, but not OA, results in decreased expression of nicotinamide nucleotide transhydrogenase (*NNT*), a key mitochondrial regulator of energy transduction and redox homeostasis, with a parallel increase of ROS and Th17 type cytokines [110]. *NNT*, whose expression is downregulated in obesity, was proposed as SFA-regulated rheostat of the redox balance that shapes T cell responses in obesity [110].

Conversely, POA was found to reduce ConA-induced T lymphocyte proliferation and to impair CD28 externalization, thus preventing the activation of transcription factors involved in inflammatory cytokine gene expression [113]. In the same study, treatment with OA results in reduced *IFN-γ* release and increased expression of anti-inflammatory factors [113]. POA and OA were also shown with T_reg_ cell differentiation, whereas stimulation of circulating T_reg_ cells with serum albumin-bound OA results in their increased survival, suggesting a role for these MUFA in maintaining immune homeostasis [113,114].

According to the effects on T lymphocyte proliferation reported in in vivo studies, EPA and DHA exposure of PBMC prior to PMA/ionomycin-induced activation was reported to reduce *IL-2* and *TNFα* but not *IFN-γ* production by *CD4*+ T lymphocytes, through the activation of *PPAR-γ* [131].

These studies widely demonstrate the capacity of FA to finely regulate and orient immune responses and suggest that selective T cell-mediated responses can be elicited through either a direct effect of FA on lymphocytes or indirect modulation of the functions of antigen presenting cells, such as a monocyte and DC.

## 5. Conclusions

**Figure 2:**
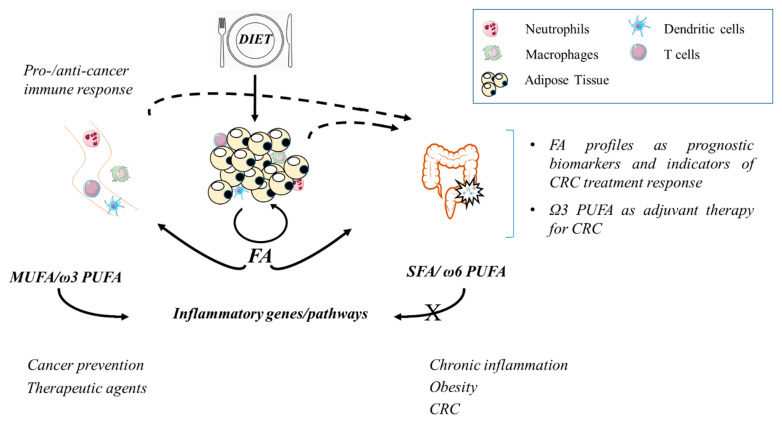
A schematic representation of dietary fatty acid (FA)-mediated regulation of adipose tissue-associated and blood immune cells. The opposite regulation of inflammatory pathways by different FA classes and the consequences on the promotion or prevention of CRC and other obesity-related morbidities are depicted.

AT-associated inflammation promoted by unhealthy dietary habits and obesity is a main mechanism that may favour CRC development and progression [136]. Inflamed AT may contribute to tumorigenesis by releasing proinflammatory mediators, including FA, and by modifying the lipid metabolism, which is part of the reprogrammed energy metabolism that characterizes cancer [137]. In turn, cancer cells have the ability to perpetuate inflammation by inducing metabolic changes in AT and to use released FA as substrates for their proliferation [39,138]. Excessive free FA or unbalanced FA profiles characterizing obesity and CRC may also affect cancer development or progression by deeply regulating gene expression in immune cell populations active in cancer surveillance (Figure 2). This is particularly relevant for CRC and other gastrointestinal cancers whose onset and evolution are profoundly influenced by the immune system.

Genes encoding cytokines, chemokines, cyclooxygenases, and matrix metalloproteinases, or regulating ROS production are major targets of FA. The pro- and anti-inflammatory actions of different FA are exerted through the triggering of membrane receptors (*TLR* and *GPR*) and the regulation of different TF, and often involve the inhibition or activation of *NF-kB* and *PPAR* family members.

Chronic activation of proinflammatory and pro-oxidant signalling pathways in innate immunity cells and the promotion of Th17 type responses, induced by SFA and ω6 PUFA, can generate, at local and systemic levels, a suppressive microenvironment favouring immune evasion and cancer growth. On the other hand, dietary intake and/or supplementation of anti-inflammatory ω3 PUFA has the potential to foster a healthy immune system for cancer control and to lower the risk of developing CRC and other inflammation-related diseases. This can have profound implications for CRC prevention, and highlights the relevance of correct dietary habits and the pivotal role of AT in the health status preservation.

Beyond the regulatory effects on the immune response, anti-inflammatory FA might play a role in CRC as preventive or therapeutic agents by targeting several genes and signalling pathways in cancer cells, and by inducing epigenetic modifications [139]. They can also influence post-diagnosis CRC outcomes by exerting beneficial effects on efficacy and tolerability of chemotherapy [140]. Finally, evidence is emerging that FA profiles have the potential to discriminate between early and late CRC stages, suggesting that they might be studied as possible prognostic biomarkers and indicators of treatment response [139,141]. However, available clinical data are still insufficient and definitive prospective randomized control trials are needed in the near future to clearly demonstrate the preventive and prognostic role of FA in CRC.

## Figures and Tables

**Table 1:** The main intervention studies evaluating the effects of fatty acid (FA) on peripheral blood mononuclear cells (PBMC) gene expression.

Dietary Compound/FA	Subjects	Treatment Duration and Doses	Genes/Pathways Targeted	TFs Involved	Reference
ω3 PUFA	Metabolically healthy overweight and obese subjects	6 weeks 3 g/day	↓Oxidative stress response	*NRF2, PPAR-α, HIF, NF-κB*	[82]
ω3 PUFA	Insulin-resistant obese subjects	8 weeks 1.8 g/day		*NRF2, PPAR-α, HIF, NF-κB*	[83]
ω3 PUFA	Obese women	3 months	↑Lipid metabolism ↑Antioxidant enzymes	*↑PPAR-α, NRF2*	[84]
FO	Alzheimer disease subjects	6 months 1.7 g/day DHA, 0.6 g/day EPA	↓Neuro-inflammation		[85]
FO	Elderly subjects	26 weeks 0.4–1.8 g/day EPA + DHA	↓Eicosanoid synthesis ↓*TLR*/Interleukin/*MAPK* signalling ↓Oxidative stress ↓Cell adhesion ↓Hypoxia signalling	↓*NF-κB* ↑*PPAR/LXR/RXR*	[86]
HOSO		26 weeks	4.0 g/day ↓Eicosanoid synthesis ↓Interleukin/*MAPK* signalling		
FO	PCOS women	12 weeks 2.0 g/day	↓*IL-1, CXCL8*	↑*PPAR-γ*	[87]
EPA + DHA	Healthy subjects with moderate hypertriglyceridemia	8 weeks 0.85 g/day and 3.4 g/day	No effects on inflammatory and endothelial function		[88]
FO	CHD patients	8 weeks	No effects on inflammatory and endothelial function		[68]
FO	Healthy lean subjects	7 weeks 8 g/day	↑ER stress response ↑Apoptosis ↑Cell cycle regulation ↑Antioxidant response	↑ 35 TF (i.e., *ATF4, MIF1, E2F, TP53, STAT1, FOXO4, SP1, NRF2*)	[89]
Krill oil	Healthy subjects	8 weeks 4 g/day	↓Glucose metabolism ↓Lipid metabolism ↓Inflammation ↓β-oxidation	↓ *SREBF2* ↓*LXRα*	[90]
HOSO		8 weeks	4 g/day ↓Lipid metabolism ↓Inflammation	↓*LXRα* ↓*PPAR-δ*	
PUFA (40% DHA)	Healthy males	6 h	↓Inflammation ↓*LXR* signalling ↑Cellular stress response	↓*LXR* ↓*SREBF1*	[91]
SFA			↑Inflammation	↑*LXR* signalling ↓Cellular stress response ↑*LXR* ↑*SREBF1*	
CSO	Impaired fasting glucose subjects	12 weeks	↓Inflammation ↓*IFNG*	*↑PPAR-γ*	[68]
ALA	Obese subject	12 weeks 4.0 g/day	↓Inflammation	*↑PPAR-γ*	[92]
Flaxseed oil	T2DM subjects with CHD	12 weeks 400 mg ALA twice a day	↓Inflammation	*↑PPAR-γ*	[93]
ω6 PUFA	Healthy subjects	8 weeks 12.9 g/day	↓Lipid metabolism (i.e., *LDLR*, *ABCG, SREBF1*, and *FASN*) ↓Inflammation (i.e., *IRAK1, TNFSF1,* *TLR4, GATA3, IL2RG, and CD8A*)	↓*PPAR-δ* ↓*LXRA*	[80]

↑ Increases; ↓ decreases; *NRF2*, nuclear factor erythroid-2-related factor-2; *PPAR-α*, peroxisome proliferator activated receptors-α; *HIF*, hypoxia incucible factor, *NF-kB*, nuclear transcription factor kappa B; *TNFα*, tumour necrosis factor α; FO, fish oil; DHA, docosahexaenoic acid; EPA, eicosapentaenoic acid; *TLR*, toll-like receptor; ER, endoplasmic reticulum; *MAPK*, mitogen-activated protein kinase; *LXR*, liver X receptor; *RXR*, retinoid X receptor; HOSO, high-oleic acid sunflower oil; *IL1β*, interleukin-1β; *CXCL8*, C-X-C motif ligand 8; *ATF4*, activating transcription factor 4; *MIF1*, macrophage migration inhibitory factor 1; *E2F*, elongation factor 2F, *TP53*, tumour protein 53; *STAT1*, signal transducer and activator of transcription 1; *FOXO*, fork-head box O; *SP1*, specificity protein 1; *SREBF*, sterol regulatory element-binding factor; PUFA, polyunsaturated fatty acids; SFA, saturated fatty acids; CSO, camelina sativa oil; IFN-γ, interferon gamma; T2DM, type 2 diabetes mellitus; CHD, coronary heart disease; PCOS, polycystic ovary syndrome; LDLR, low-density lipoprotein receptor; *ABCG*, ATP binding cassette-G; *FASN*, fatty acid synthase; *IRAK1*, interleukin-1 receptor-associated kinases; *TNFSF1*, tumour necrosis factor superfamily 1; *TLR4*, toll-like receptor 4; *GATA3*, GATA-binding protein 3; *IL2RG*, IL2 receptor common gamma chain.

**Table 2:** In vitro studies evaluating the immunomodulatory effects of fatty acid (FA) on immune cells.

Dietary Compound/ FA	Cell Type	Downstream Effects	Pathway(s) Targeted	TFs Involved	Reference
***SFA***					
PA	THP-1 Primary monocytes	↑*IL-1, IL-6, IL-18, TNFα, CCL2, CCL4, CXCL8*	↑*TLR4/MyD88/MAPK*	↑*NF-kB, AP-1*	[98,99,100,101]
PA (in combination with TNFα)	THP-1 Primary monocytes/macrophages	↑*CCL2, CCL3, CCL4, CXCL8*	↑*TLR4/TRIF*	↑*NF-kB, AP-1, IRF3*	[102,103,104]
PA	THP-1	↑*IL-1β*	↑*TLR2/TLR1* ↑*NLRP3* inflammasome		[105]
PA	MDDC	↑*IL-1β* ↑ROS ↑Activation/maturation	↑*TLR4/MD-2* ↑*NLRC4* Inflammasome	↑*NF-kB*	[106]
SFA	MDDC	No effects on inflammation			[107,108]
OA, LA, GLA	Neutrophils	↑ROS			[109]
PA	PBMC	↓*NNT* ↑ROS ↑Th17-type cytokines			[110]
PA	Naïve T lymphocytes	↑*SLAMF3*	↑*PI3K/AKT*	↑*STAT5*	[111]
***MUFA***					
OA	Neutrophils	↑Phagocytosis and killing ↑ROS ↑*IL-1β, CXCL3, VEGF*	↑Intracellular calcium ↑*PKC*		[109,112]
OA	T lymphocytes	↓*IFNγ* ↑*IL-4, IL-10*			[113]
POA		↓ConA-induced T lymphocyte proliferation	↓Treg cell differentiation ↓*IL-2, IL-6, IFNγ, TNFα, IL-17A* ↓CD28 externalization	↓*NFAT, AP-1, NF-κB*	
Albumin-bound OA	PBMC-sorted Treg cells	↑Treg cell survival			[114]
***ω6 PUFA***					
AA	Neutrophils	↑ROS ↑*TNFR1, TNFR2*	↑Intracellular calcium ↑*PKC, ERK1/2, cPLAP2*		[112,115]
ω6 PUFA	Neutrophils	↓ATP ↑LDH release ↑Mitochondria depolarization			[116,117]
AA	THP-1 Primary monocytes		↓*IFNγ* signalling pathway ↓*IDO*	↓*STAT1* phosphorylation	[118]
AA	Differentiating MDDC	↓*CD40, CD80, MR* ↓LPS-induced *CD40, CD80, CD83, CD86* ↓*IL-12p40, TNFα* ↓T cell proliferation ↓*IL-2/IFNγ* in co-cultured T cells		*NF-κB* independent	[108]
A1AT-LA	Neutrophils	Anti-inflammatory	↓*IL-1β*	↑*PPAR-γ*	[119]
***ω3 PUFA***					
EPA/DHA	THP-1	↓*IL-1β, IL-18, TNFα, PAI-1*		*↓NF-κB*	[62,100,120,121]
EPA/DHA	U937	M2 polarization	↑*p38 MAPK*	*↑KLF4*	[122]
EPA/DHA	THP-1	↓LPS-induced cytokine gene expression (i.e., *IL6, TNFα, IL1β, MCP1, TNFAIP3,* and *PTGS2*)			[123]
DHA	MDM	Anti-inflammatory	Via *GPR120* ↑*cPLAP2* ↑*PGE2* signalling		[120]
EPA/DHA	THP-1	Anti-inflammatory	Via *GPR120/GPR40/β*-Arrestin-2 ↓*NLRP3* inflammasome		[62,124]
EPA/DHA	MDDC	↓Cell activation and cytokine production ↓*MHC-II, CD40, CD80, CD86, CD83* ↓*TNFα, IL-10, IL-12* ↓*IL-2/IFNγ* in co-cultured T cells ↓T cell stimulation	↓*p38 MAPK*	↑*PPAR-γ*	[107,125]
DHA	Differentiating MDDC	↑*MHC-II* ↓*IL-12p70, IL-6, IL-10* ↓T cell stimulation		*PPAR:RXR*	[126]
EPA/DHA/ ALA	MDDC	↓*CD1a*+ cells ↓*IL-6*	↓*GPR120*		[127]
EPA	Differentiating MDDC	↓*CD40, CD80, MR* ↓LPS-induced *CD40, CD80, CD83, CD86* *↓IL-12p40, TNFα* ↓T cell proliferation ↓*IL-2/IFNγ* in co-cultured T cells		*NF-κB* independent	[108]
Oxidized EPA	Monocytes Neutrophils	↓Adhesion to endothelial cells		↑*PPAR-α*	[128]
ω3 PUFA	Neutrophils	↑Chemotaxis, ROS production, NADPH-oxidase activation	↑*GPR84*		[129]
OA/ALA/DHA	PBMC	↓LPS-induced inflammatory genes (*IL-6, TLR2, TNFα, COX2*)		↓*NF-κB*	[130]
EPA/DHA	PBMC	↓CD4+ T lymphocyte produced *IL-2*, *TNFα, IL-4*		↑*PPAR-γ*	[131]

↑ Increases; ↓ decreases, SFA, saturated fatty acids, PA, *IL-1β*, interleukin-1β; *IL-6*, interleukin-6; *IL-18*, interleukin-18; *TNFα*, tumour necrosis factor α; *TLR4*, toll-like receptor 4; *APK*, /*TRIF*, Toll/IL-1 receptor (TIR) domain-containing adaptor; *MD-2*, myeloid differentiation factor 2; *NF-kB*, nuclear transcription factor kappa B; *AP1*, activator protein 1; *IRF3*, interferon regulatory factor 3; *PAI-1*,plasminogen activator inhibitor-1; *NLRC4*, NLR family CARD domain containing 4; *NLRP3*, NOD-like receptor family, pyrin domain containing 3; ROS, reactive oxygen species; *NNT*, nucleotide nicotinamide transhydrogenase; *SLAMF3*, signalling lymphocytic activation molecule family 3; *STAT*, signal transducer and activator of transcription; *VEGF*, vascular endothelial growth factor; *NFAT*, nuclear factor of activated T cells; MUFA, monounsaturated fat acids; OA, oleic acid; ALA, α-linoleic acid; LA, linolenic acid; GLA, γ linolenic acid; PUFA, polyunsaturated fatty acids; POA, palmitoleic acid; AA, arachidonic acid; *A1AT*, alpha1-antitrypsin; DHA, docosahexaenoic acid; EPA, eicosapentaenoic acid; *TNFR*, tumour necrosis factor receptor; *IDO*, indoleamine 2,3-dioxygenase; *MCP1*, macrophage inflammatory protein-1; *TNFAIP3*, TNF-alpha-induced protein 3; *PTGS2*, prostaglandin-endoperoxide synthase 2; *GPR120*, G protein-coupled receptor 120; *cPLAP2*, plastid-lipid-associated proteins; *PGE2*, prostaglandin E2; *GPR120/GPR40*, G protein-coupled receptor 120/40; *MHC-II*, major histocompatibility complex class II; *p38 MAPK*, p38 mitogen-activated protein kinase; *KLF4*, Kruppel-like factor 4; *PPAR*, peroxisome proliferator activated receptors; *RXR*, retinoid X receptors; MDDC, monocyte-derived dendritic cells; MDM, monocyte-derived macrophages; *NNT*, nicotinamide nucleotide transhydrogenase.

## Data Availability

No new data were created or analysed in this study. Data sharing is not applicable to this article.
